# Unusual scalp crusted scabies in an adult T-cell leukemia/lymphoma patient

**DOI:** 10.3109/03009734.2010.548877

**Published:** 2011-02-11

**Authors:** Yi-Chun Lai, Chung-Jen Teng, Paul Chih-Hsueh Chen, Tzeon-Jye Chiou, Chun-Yu Liu

**Affiliations:** ^1^National Yang-Ming University School of Medicine, TaiwanRepublic of China; ^2^Department of Medicine, National Yang-Ming University Hospital, Ilan, TaiwanRepublic of China; ^3^Division of Hematology/Oncology, Department of Medicine Taipei Veterans General Hospital, Taipei, TaiwanRepublic of China; ^4^Division of Transfusion Medicine, Department of Medicine Taipei Veterans General Hospital, Taipei, TaiwanRepublic of China; ^5^Department of Pathology and Laboratory Medicine, Taipei Veterans General Hospital, Taipei, TaiwanRepublic of China

**Keywords:** Adult T-cell leukemia/lymphoma, crusted (Norwegian) scabies, human T-cell lymphotropic virus 1

**Figure 1.**
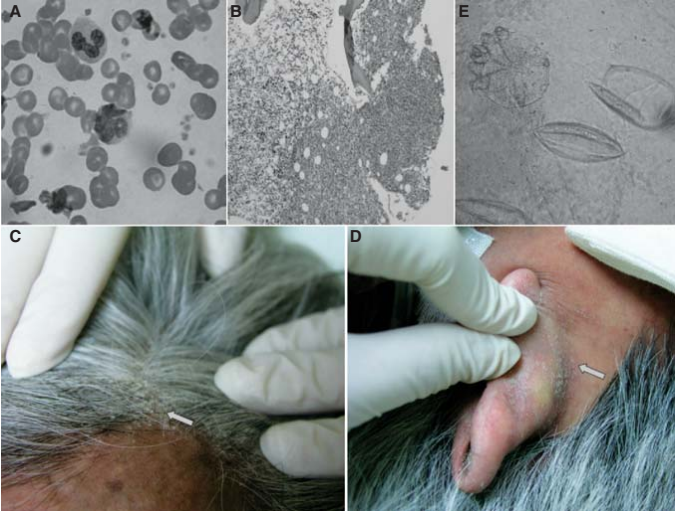
A: A flower-like nucleated T cell in the peripheral blood smear from the patient. B: Pathology of marrow biopsy revealed lymphomatous involvement in marrow space. C, D: Crusted scaly lesions at the scalp and posterior auricular skin fold (arrow). E: Microscopic examination of a scraping scale shows two hatched eggs (right) and a mite (left) (KOH, ×400).

A 54-year-old Taiwanese lady presented with intermittent fever for 1 week. Examination revealed hepatosplenomegaly. Laboratory studies revealed marked leukocytosis (leukocyte count of 316,000 /μL) and an elevated lactate dehydrogenase of 2,929 IU/L. Examination of her peripheral blood morphology disclosed abnormal lymphoid cells with flower-shaped nuclei (Figure 1A), and a subsequent serology testing for human T-cell lymphotropic virus 1 (HTLV-1) antibody showed a positive result. A bone-marrow biopsy specimen demonstrated marrow infiltration of atypical lymphoid cells (Figure 1B), around 20%–30%, which were immunoreactive for UCHL-1, CD3, and CD7, but non-immunoreactive for CD20, CD34, TdT, and myeloperoxidase. In addition, multiple intra-abdominal lymphadenopathies were discovered by a computed tomography scan. She was diagnosed with acute type adult T-cell lymphotropic/leukemia (ATL) and was treated with a combination of chemotherapy regimen (cyclophosphamide, vincristine, doxorubicin, and prednisolone (CHOP)), isotretinoin (Roaccutane), and subcutaneous recombinant interferon alfa-2a (Roferon-A). A response of partial remission was achieved after the treatments, and her following blood routines were in stable status. About 14 months later, she came to our emergency room because of general malaise and abdominal fullness for several days. She had a leukocyte count of 19,400/μL and hypercalcemia (free calcium of 2.57 mmol/L) on laboratory investigation. Moreover, she presented with scaly crusted skin lesions over the scalp (Figure 1C) and external ears (Figure 1D), which were initially considered as skin involvement of ATL. No burrows were identified between fingers or over wrists or other skin parts. However, microscopic examination of the scraping scales with potassium hydroxide staining disclosed many scabies mites and hatched eggs (Figure 1E). Norwegian scabies was settled and she was treated with topical anti-scabies ointment, gamma benzene hexachloride. Unfortunately, her condition deteriorated rapidly with development of sepsis and subsequent acute respiratory distress syndrome. She died of rapid progression of ATL 1 week later.

HTLV-1 infection is not endemic in Taiwan (1); the prevalence of ATL in Taiwan has been reported to be 6% in 317 patients with non-Hodgkin's lymphoma (NHL) during 1983–1988 in northern Taiwan (2), and 2.8% in 72 patients with T-cell NHL during 1989–2002 in southern Taiwan (3). In HTLV-1 non-endemic regions, the diagnosis of ATL can be challenging and difficult to establish.

Crusted scabies has been reported in HTLV-1-seropositive patients, some of whom had ATL (4,5). The scabies skin lesions usually involve limbs and trunk, either localized or diffuse, and only rarely involve the face or scalp area (6,7). The dermatological presentation of scabies seen in our patient was unusual in that only the scalp and ears were predominately involved, and there were no classical ‘burrows’ that were commonly seen on hands and wrists in patients with scabies infections. HTLV-1-induced immunosuppression has been linked to the occurrence of crusted scabies in HTLV-1 carriers and in ATL patients (8). The atypical dermatological presentation of scabies in patients with ATL may cause diagnostic confusion with the more commonly seen skin involvement of ATL. Moreover, the presence of scabies in ATL patients may represent a sign of marked immunosuppression and thus indicate a poorer prognosis. The rapid deterioration after the diagnosis of scabies seen in our patient supports this correlation. Although ATL-associated skin lesions are common in ATL patients, crusted scabies should also be considered in the differential diagnosis of either localized or generalized cutaneous eruptions in patients with ATL.
